# An Audit of Sexual Health Screening Practices on the General Adult Female Inpatient Wards in Mersey Care NHS Foundation Trust

**DOI:** 10.1192/bjo.2025.10606

**Published:** 2025-06-20

**Authors:** Lily Farakish, Tasnim Elbadrawy, Ruchi Desai, Declan Hyland

**Affiliations:** Mersey Care NHS Foundation Trust, Liverpool, United Kingdom

## Abstract

**Aims:** Women with mental illness face increased sexual and reproductive health risks, including trauma, sexually transmitted infections (STIs), reproductive-associated diseases, and contraceptive challenges. In 2024, Mersey Care NHS Foundation Trust ratified its clinical policy for physical health care. The authors assessed compliance with annual physical health checks (APHCs), sexual health assessments, and their communication in discharge summaries. The aims of this audit were to identify gaps and develop improvement strategies for the Trust’s general adult female inpatient wards.

**Methods:** A retrospective audit design was used with convenience sampling based on the data collection day. Compliance was benchmarked against NICE guidelines and the Trust’s Clinical Policy SD29 (target 100%). Data were extracted from electronic patient records for APHCs, sexual and reproductive health screenings, and evidence of actionable findings in discharge summaries. If no valid APHC was available, the most recent assessment was searched for. Topics were “covered” if decision-making processes were documented.

**Results:** Sixty-two female patients from four general adult inpatient wards were identified. Mean age was 42 years (19–70 years). Mean admission length was 50.82 days (1–339 days). Mental health diagnoses included schizoaffective, psychotic, anxiety, depressive, neurodevelopmental, and personality disorders. 60% of inpatients were detained under Section 2 of the Mental Health Act 1983, 19% under Section 3, and 21% were voluntary.

Regarding APHCs:

94% valid (none fully complete).

Four patients without a valid form; two had documented reasons and one offered follow-up.

All national cancer screening programmes were covered in 55% of age-eligible patients (6% declined).

55% covered sexual health history (3% declined).

22% covered menstrual history.

12% covered obstetrics and gynaecology history (3% declined).

Regarding inpatient screening:

90% lacked documented contraceptive discussions beyond oral contraception on admission.

Pregnancy tests: 48% on admission, 15% during admission, 13% documented reason for non-completion; 25% non-completion in over 45s likely due to undocumented menopausal status.

STI screening considered in 13% of cases.

Sexual health counselling offered to 11%. Four out of seven inpatients accepted.

One patient had a positive STI screen in a previous admission.

**Conclusion:** Despite high APHC completion rates, gaps in sexual health screening and in documentation were identified. Key improvements include better integration of sexual health within assessments, increased review of reproductive health needs, and more consistent STI screening. The authors suggest exploring staff awareness, regional staff teaching, updating policy guidance, and increasing policy distribution. Future audits should evaluate intervention effectiveness, ensuring comprehensive care for this vulnerable patient population.

